# New Horizons in Psychiatry

**Published:** 1897-11

**Authors:** 


### Abstracts and Selections.

From the *New York Medical Record's* cabled report of the International Medical Congress at Moscow we extract the following:

NEW HORIZONS IN PSYCHIATRY.—Prof. Cesare Lombroso, of Turin, was the last orator. When he stepped forward on the platform he was received with long-continued and repeated rounds of applause, just as his name was cheered to the echo when it was read, on the first day of the congress, among those of the national presidents. The doctrine of criminal anthropology, of which the Turin professor is the most distinguished apostle, numbers many of its most enthusiastic and active supporters among Russian men of science. In the introduction to his subject the speaker said that by the term "new horizons" he meant new applications of the science of psychiatry, namely, the application of the facts of this branch of learning to a study of abnormal man, of the criminal and degenerate. All new sciences meet with great opposition at first, and it is best that they should. This new science had not escaped the usual fate; it had even been denied the distinction of being a science. But there is, the speaker maintained, much true, earnest, scientific work being done in it. The tendency of the science is not to undermine the penal code, as some legislators have imagined, but its aim is solely to protect diseased humanity; it is to turn society from its cherished system of vengeance for wrongs done to it by irresponsible beings, to a policy of correction and of prevention of crime. Unfortunately, and this is a fact which has been used by opponents to discredit the science of criminal anthropology, pathology is of little or no help in the study. But there is the same drawback in the study of many nervous diseases, for in perhaps the majority of them, the so-called functional nervous diseases, there are no definite lesions discoverable with the aids at our command. There is hope, however, for the future in the new theory of the neuron. In this theory of independent movements of the ueurons we find a very satisfactory explanation of the phenomena of sleep, of hypnotism and of memory. The fact that in dealing with supposed psychical states we are dealing with pure hyphothesis is one that tends to discourage observation, but it should not. We ought to study carefully all modifications of the psychical state, for the discoveries which this may lead us to are incalculable in their importance. An explanation of telepathy or mental influence acting at a distance is possibly to be found in a polarization of the molecular elements of the brain substance. This science, this study of psychiatry, which has been but begun in the nineteenth century, will be handed down as a legacy to the twentieth, and the development which it may attain and the benefits to the human race which may flow from its discoveries are beyond our powers to foresee—indeed, beyond even the powers of imagination.

